# CA19-9 as a Potential Target for Radiolabeled Antibody-Based Positron Emission Tomography of Pancreas Cancer

**DOI:** 10.1155/2011/834515

**Published:** 2011-09-06

**Authors:** Mark D. Girgis, Tove Olafsen, Vania Kenanova, Katelyn E. McCabe, Anna M. Wu, James S. Tomlinson

**Affiliations:** ^1^Department of Surgery, UCLA, 10833 LeConte Avenue, Rm 54-140, Los Angeles, CA 90095, USA; ^2^Department of Surgery, Veterans Affairs Greater Los Angeles, Health Care Center, Los Angeles, CA 90073, USA; ^3^Department of Molecular and Medical Pharmacology, Crump Institute for Molecular Imaging, Los Angeles, CA 90095, USA

## Abstract

*Introduction*. Sensitive and specific imaging of pancreas cancer are necessary for accurate diagnosis, staging, and treatment. The vast majority of pancreas cancers express the carbohydrate tumor antigen CA19-9. The goal of this study was to determine the potential to target CA19-9 with a radiolabeled anti-CA19-9 antibody for imaging pancreas cancer. *Methods*. CA19-9 was quantified using flow cytometry on human pancreas cancer cell lines. An intact murine anti-CA19-9 monoclonal antibody was labeled with a positron emitting radionuclide (Iodine-124) and injected into mice harboring antigen positive and negative xenografts. MicroPET/CT were performed at successive time intervals (72 hours, 96 hours, 120 hours) after injection. Radioactivity was measured in blood and tumor to provide objective confirmation of the images. *Results*. Antigen expression by flow cytometry revealed approximately 1.3 × 10^6^ CA19-9 antigens for the positive cell line and no expression in the negative cell line. Pancreas xenograft imaging with Iodine-124-labeled anti-CA19-9 mAb demonstrated an average tumor to blood ratio of 5 and positive to negative tumor ratio of 20. *Conclusion*. We show *in vivo* targeting of our antigen positive xenograft with a radiolabeled anti-CA19-9 antibody. These data demonstrate the potential to achieve anti-CA19-9 antibody based positron emission tomography of pancreas cancer.

## 1. Introduction

Pancreatic cancer is one of the most lethal cancers where incidence approximates mortality [1]. Symptoms of pancreatic cancer are vague and usually occur late in the disease process. As a result, 80% of all pancreas cancer patients have metastatic disease at diagnosis [2] leading to poor patient outcomes with overall actual 5-year survival of approximately 6% [3]. Even for the 10–20% who undergo surgical resection, overall 5-year survival is only 25% due to the presence of small foci of metastatic disease in the lymph nodes or liver that are unrecognized by our current imaging (CT/MRI/FDG-PET) [4–6]. These data indicate that patients are routinely understaged and underscore the need for improved imaging techniques to accurately detect the true burden of disease. 

Molecular imaging shows great promise to provide sensitive and tumor-specific imaging. The most commonly used molecular imaging modality is positron emission tomography (PET). It has been estimated that PET technology has the potential to provide highly sensitive imaging based on its ability to detect nanomolar to subnanomolar concentrations of radiolabeled imaging agents and provide 1-2 mm spatial resolution [7]. Most often in the clinical setting, PET scanning technology uses 2-deoxy-2-[^18^F]fluoro-D-glucose (FDG) as the imaging agent, taking advantage of a tumor cell's metabolic dependence on glycolysis (the Warburg effect), which results in increased glucose uptake relative to nonmalignant cells. Although FDG-PET has shown great potential to image many cancers, it has limited utility in pancreas cancer secondary to a lack of sensitivity and specificity [8–12]. With the introduction of radiolabeled antibodies and antibody fragments as PET imaging agents, immunoPET exploits the inherent sensitivity of PET to provide tumor-specific imaging. Specifically, monoclonal antibodies (mAbs), which offer high specificity for epitopes known to be differentially expressed on cancer cells, can be labeled with a positron emitting radionuclide to provide antigen-specific PET images. 

With respect to pancreas cancer, CA19-9 is the most highly expressed tumor antigen, present on cellular membrane proteins in more than 90% of patients [13–17]. This tumor antigen is minimally expressed in normal pancreas epithelial [18, 19]. The molecular mechanism responsible for the increased expression of CA19-9 in pancreas cancer is secondary to aberrant glycosylation of proteins upon malignant transformation of epithelial cells [20–22]. The relatively high expression of CA19-9 on membrane proteins of the pancreas cancer cells represents an attractive target for the development of pancreas cancer-specific targeting agents. Moreover, since the CA19-9 epitope can be present in multiple copies on a single membrane protein, the potential of being able to target more than one epitope per membrane protein exists, giving CA19-9 a theoretical advantage over other protein tumor antigens like CEA. Thus, we decided to investigate the usefulness of CA19-9 as a tumor target for pancreatic cancer immunoPET. 

Our first objective was to develop and characterize a preclinical xenograft model which recapitulates the human condition of pancreatic cancer with respect to CA19-9 expression. To this end, we evaluated and quantified CA19-9 expression on our human pancreatic cancer cells by flow cytometry and performed immunohistochemical staining of our xenografts to compare to human cases of pancreas cancer. Finally, we tested our ability to obtain antigen-specific PET imaging of our pancreas cancer xenografts utilizing a radiolabeled anti-CA19-9 monoclonal antibody.

## 2. Methods

### 2.1. Production of Anti-CA19-9 Antibody

Hybridoma cells (1116-NS-19-9) that secrete the intact mouse monoclonal anti-CA19-9 antibody were purchased from the American Type Culture Collection (ATCC, Manassas, VA). These cells were maintained as recommended by the ATCC and supplemented with 1% penicillin/streptomycin (Invitrogen, Carlsbad, CA). Cells were grown and expanded into triple flasks (Nunclon, Rochester, NY). Supernatants from terminal cultures were harvested from triple flasks, centrifuged to remove cell debris, and sterile filtered. Proteins from the supernatant were purified using affinity chromatography with a 1 mL Protein G column (Applied Biosystems, Carlsbad, CA) on an AKTA Purifier (GE Healthcare, Piscataway, NJ). Bound protein was eluted at 30% 0.2 M citric acid buffer, pH 2.1 and phosphate buffered saline (PBS). Eluted proteins were immediately brought back to neutral pH with 1 M Tris-buffer, titrated to pH 8.2 with 6 M NaOH. All fractions were analyzed by sodium dodecyl sulfate polyacrylamide gel electrophoresis (SDS-PAGE). Fractions containing the protein of interest were pooled, and dialyzed against PBS using a Slide-A-Lyzer Dialysis Cassette, molecular weight cutoff 30,000 daltons (Thermo Fisher Scientific, Rockford, IL). Pure protein was then concentrated by Vivaspin 20, molecular weight cutoff 30,000 daltons (Thermo Fisher Scientific), and the final concentration was determined by A_280 nm_ using an extinction coefficient of *ε* = 1.4.

### 2.2. Characterization of Purified Antibody

Purified antibody was analyzed by SDS-PAGE on pre-cast 4–20% polyacrylamide Ready Gels (Bio-Rad Laboratories, Hercules, CA) under reducing (1 mmol/L DTT) and nonreducing conditions. The proteins were detected by staining with Microwave Blue (Protiga Inc., Frederick, MD). Western blot using alkaline phosphatase (AP) conjugated goat antimouse IgG, Fc specific, (Jackson ImmunoResearch Labs, West Grove, PA) antibody (1 : 5000) was used to confirm the results seen on SDS-PAGE. Western blots were developed with 33 *μ*L of standard stock solution of BCIP (5-bromo-4-chloro-3′-Indolyl phosphate p-toluidine salt, Bio-Rad Laboratories) and 66 *μ*L of standard stock solution of NBT (nitro-blue tetrazolium chloride, Bio-Rad Laboratories) in 10 mL of AP buffer. Samples were also subjected to size exclusion chromatography (SEC) on a Superdex 200 HR 10/30 column (GE Healthcare, Piscataway, NJ) run isocratically in PBS. A 100 *μ*L volume containing 50 *μ*g of pure protein was loaded onto the column and eluted with PBS at a flow rate of 0.5 ml/min. Elution time was compared to bovine serum albumin (66 kDa) and *β*-amylase (200 kDa) standards (Sigma). The anti-CA19-9 antibody dissociation constant (i.e., binding affinity) was determined by cell-based ELISA. Cells were harvested, and approximately 5 × 10^4^ were transferred to each well of a 96-well round bottom plate (Costar, Corning, NY) and incubated overnight. The following day each well was washed with 150 *μ*L of PBS and incubated with increasing concentrations of antibody (0.01–100 nM). After one hour incubation, the wells were washed and incubated for another hour with a fixed concentration (1 : 1000) of AP-conjugated goat anti mouse IgG, Fc specific (Jackson ImmunoResearch) antibody. The cells were washed again and developed with phosphatase substrate tablets (Sigma) dissolved in diethanolamine buffer, pH 9.8. All assays were carried out in triplicate.

### 2.3. Antigen Expression and Quantitation

The expression of CA19-9 was determined for each cell line by flow cytometry. For each cell line, 1 × 10^6^ cells were harvested from culture and resuspended in 250 *μ*L of Phosphate Buffered Saline/1% Fetal Bovine Serum (PBS/1%FBS). Purified intact mouse anti-CA19-9 antibody was added in excess (4 *μ*g) and incubated for 1 hour. The samples were centrifuged at 1000 g for 10 minutes, and the supernatant was discarded. Following a wash step, the samples were each resuspended in 250 *μ*L of PBS/1% FBS. Four micrograms of the secondary antibody, phycoerythrin (PE) conjugated, goat antimouse IgG (Fc specific), antibody (Jackson ImmunoResearch Labs), were then added to each sample and incubated for 1 hour. After a final wash step, cells were resuspended in PBS/1% FBS and the fluorescence associated with the cells was measured. Negative controls included samples with cells only and samples with only secondary antibody. Quantitation of antigen expression for each cell line was performed using the Dako Qifikit according to the manufacturer's instructions (DAKO, Carpinteria, CA) using fluorescein (FITC) conjugated, goat anti mouse IgG (Fc specific) antibody. Antigen expression is referred to as antigen density and recorded as antigens per cell. All assays were performed in triplicate.

### 2.4. Immunohistochemistry

Surgically resected human pancreas cancer specimens were provided by the Department of Pathology at the University of California, Los Angeles (UCLA) Medical Center under an approved Institutional Review Board (IRB) protocol. These specimens were evaluated by IHC for expression of CA19-9 using the monoclonal mouse anti-CA19-9 antibody. Each paraffin embedded specimen was deparaffinized and incubated with the primary anti-CA19-9 antibody (1 : 50) for 1 hour. Specimens were washed with PBS/1% Tween and then incubated with the secondary horseradish peroxidase (HRP) conjugated goat anti mouse IgG (Fc specific) antibody (1 : 400) (DAKO). Negative control slides were incubated with the secondary antibody only.

### 2.5. Cell Lines

The human pancreatic cancer cell lines, BxPC3 and MiaPaca-2, were purchased from the ATCC and maintained in RPMI-1640 medium and DMEM, respectively. All media were supplemented with 100 units of penicillin and 100 *μ*g of streptomycin (Invitrogen) and 10%FBS. DMEM for MiaPaca-2 was also supplemented with 2.5% horse serum (Invitrogen).

### 2.6. Radioiodination

Radioiodination with the positron emitting isotope ^124^I was performed by the Iodo-Gen method as described [23]. The labeling reaction (0.1 ml) contained 0.1–0.2 mg purified protein and 0.5–1 mCi Na ^124^I (IBA Molecular North America, Dulles, VA). Labeling efficiency was measured by instant TLC using the Tec-Control kit (Biodex Medical Systems, Shirley, NY). Immunoreactivity was determined by incubating the radioiodinated antibody (*≈*100,000 cpm) with BxPC3 cells in PBS/1% FBS such that there was an excess of antigen present. The radioiodinated antibody was allowed to incubate for 1 hour before the samples were washed with PBS/1%FBS. Any radioactivity not bound to cells was collected in the supernatant and measured in a gamma counter (Wizard 3′′ 1480 Automatic Gamma Counter, Perkin-Elmer, Covina, CA). This fraction was divided by the total known amount of radioactivity added to the cells to yield the portion of unbound antibody. The amount of unbound antibody was subtracted from 1 to yield the portion of bound antibody (Immunoreactivity).

### 2.7. Xenograft Imaging and Biodistribution Studies

All animal handling was done under a protocol approved by the Chancellor's Animal Research Committee of the University of California in Los Angeles (UCLA). Mouse xenografts were created by injecting subcutaneously 1 × 10^6^ of antigen-negative (MiaPaca-2) and antigen-positive (BxPC3) cells into the right and left shoulders of 8-week-old, female nude mice, respectively. Xenografts were grown for approximately 3 weeks. Stomach uptake was blocked by performing gastric lavage with 1.5 mg of potassium perchlorate in 0.2 mL of PBS 30 minutes prior to injection. Thyroid uptake was not blocked. Each mouse was injected with an equal volume of the radiolabeled antibody from the radioiodination reaction batch. This ensured that each mouse received a similar amount of radiolabeled antibody. Mice were injected with approximately 35 *μ*g of ^124^I-anti-CA19.9 antibody (specific activity of 3.1 ± 1.7 *μ*Ci/*μ*g) in PBS via the tail vein. At 72, 96, and 120 hours postinjection, the mice were anesthetized using 2% isoflurane, placed on the microPET bed, and imaged with a Focus microPET scanner (Concorde Microsystems Inc., Knoxville, TN). Acquisition time was 10 minutes. All images were reconstructed using an FBP algorithm and displayed by the AMIDE software package [24, 25]. After the final microPET imaging time point, selected animals were also imaged by microcomputed tomography (microCT) for anatomic reference and subsequent coregistration with microPET images. Following the last scanning time point, animals were euthanized; tumors, blood, kidneys, liver, spleen, stomach, pancreas, lungs, and carcass were harvested and weighed. Radioactive uptake of organs was counted in a gamma counter (Wizard 3′′ 1480 Automatic Gamma Counter, Perkin-Elmer, Covina, CA) for biodistribution analysis. After decay correction, radioactive uptake in organs was converted to percentage of injected dose per gram tissue (%ID/g).

### 2.8. Serum CA19-9 Measurement

Serum CA19-9 level was performed for mice bearing antigen-positive xenografts alone and antigen-negative xenografts alone. Four nude mice were injected s.c. with 1 × 10^6^ cells. Two mice harbored the MiaPaca-2 xenograft and the other two harbored the BxPC3 xenograft. Xenografts were permitted to grow for 3 weeks. After sacrificing the animals, heart puncture was performed to obtain blood samples. Using the Cancer Antigen CA19-9 ELISA test kit (Panomics, Fremont, CA) and following the manufacturer's instructions, blood samples were tested for CA19-9 levels based on standards provided by the kit. For each animal, 3 blood samples were obtained and analyzed for CA19-9 levels, totaling 6 samples for animals with CA19-9 positive tumors and 6 samples for CA19-9 negative tumors.

## 3. Results

### 3.1. Production and Biochemical Characterization of the Anti-CA19-9 Antibody

**Figure 1:**
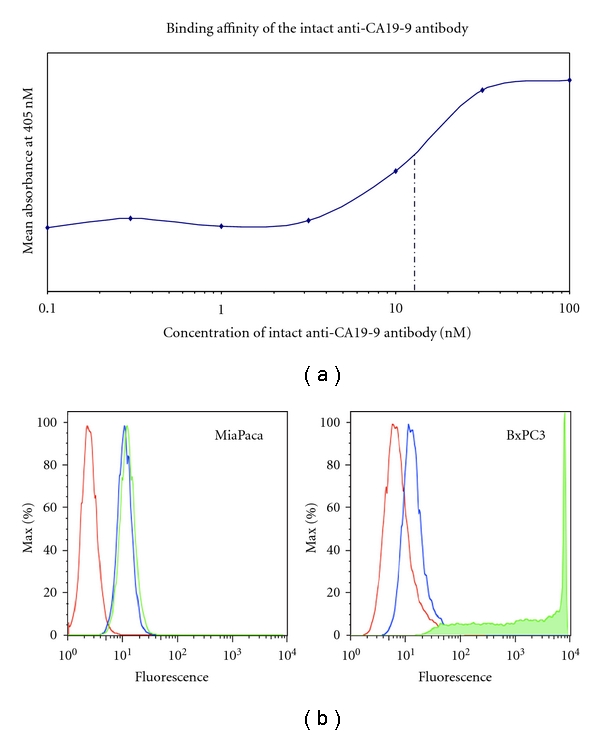
*In vitro* functional characterization of the anti-CA19-9 intact murine antibody. (a) Equilibrium binding affinity constant as determined by cell-based ELISA of the anti-CA19-9 antibody. (b) Flow cytometry showing binding specificity of the anti-CA19-9 antibody to each human cancer cell line. *Red line* represents cells only. *Blue line represents *cells incubated with only PE-conjugated goat antimouse antibody. *Green line* represents cells incubated with intact mouse anti-CA19-9 antibody and PE-conjugated goat antimouse antibody.

The level of antibody production of hybridoma cells following expansion in triple flasks was approximately 20 *μ*g/ml. SDS-PAGE under reducing and nonreducing conditions was used to confirm the purity of the anti-CA19-9 antibody after purification. The migration of the anti-CA19-9 antibody was consistent with the known molecular weight of intact antibodies (approx. 150 kDa). Reducing conditions yields the two expected fragments corresponding to the heavy and light chains of the antibody. Western blot probing for the murine Fc region confirmed protein identity (data not shown). Size exclusion chromatography shows that the intact anti-CA-19-9 monoclonal antibody elutes between the BSA (66 kDa) and *β*-amylase (200 kDa) standards at 25.7 minutes consistent with published results for IgG1 subtype antibodies [26]. Cell-based ELISA estimated the approximate equilibrium binding affinity constant (*K*_*D*_) of the anti-CA19-9 antibody to be in the range of 12–15 nM (Figure 1(a)).

### 3.2. Antigen Expression and Quantitation

Flow cytometry confirmed high expression of CA19-9 on the human pancreatic cancer cell line, BxPC3, and very low expression on MiaPaca-2 (Figure 1(b)). Quantitative flow cytometry showed that the BxPC3 cell line exhibited approximately 1.3 × 10^6^ CA19-9 epitopes per cell, while the MiaPaca-2 showed less than 400 epitopes per cell.

### 3.3. Immunohistochemistry

**Figure 2:**
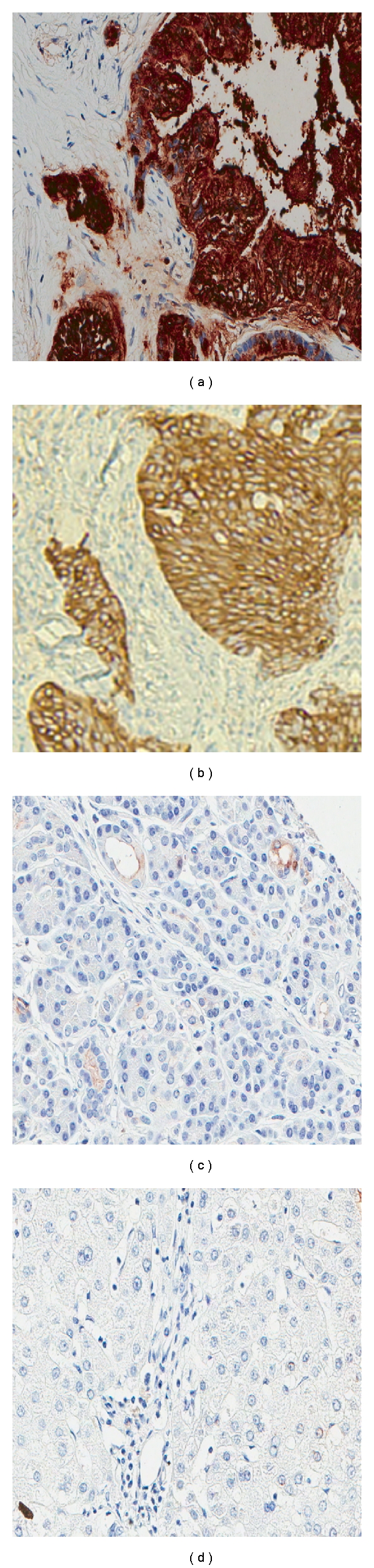
Immunohistochemistry with the anti-CA19-9 antibody (magnification 40x). (a) Human pancreas cancer specimen. (b) BxPC3 xenograft. (c) Normal human pancreas section. (d) Normal human liver section.

Expression of CA19-9 on human pancreas cancer specimens from surgically resected tumors was evaluated using IHC. Of the fourteen human pancreatic adenocarcinoma specimens evaluated, 12 were positive for CA19-9 expression yielding an 86% positivity of the small sample of human tumors that we evaluated (Figure 2). Normal liver and pancreas specimens showed little or no staining confirming low to absent expression in these normal tissues. Anti-CA19-9 IHC staining was noted to be of similar intensity between human pancreas cancer specimens and mouse xenografts specimens.

### 3.4. Radioiodination, Xenograft Imaging, and Biodistribution Studies

**Figure 3:**
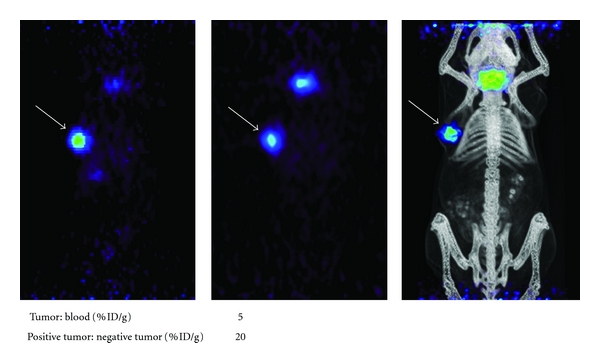
*In vivo *MicroPET images of BxPC3 xenografts. (a) Image at 72 hours. (b) Image at 96 hours. (c) Coregistered microPET/microCT Image at 120 hours. White arrows point to the BxPC3 tumor on the left shoulder of the mouse. Tumor to blood and positive tumor to negative tumor ratios of %ID/g determined from biodistribution data acquired at the 120 hour time point are indicated below the images.

Radioiodination with ^124^I was conducted with a labeling efficiency of 49%. Immunoreactivity of the radiolabeled fraction was 60%. For animal studies, microPET was employed to evaluate *in vivo* antigen-specific tumor targeting ability of the anti-CA19-9 antibody. Three nude mice, each harboring a CA19-9 positive tumor (BxPC3) and CA19-9 negative tumor (MiaPaca-2) were injected via the tail vein with approximately 35 *μ*g of ^124^I labeled anti-CA19-9 antibody. Each injection was approximately 120 *μ*Ci of radioactivity. Average tumor weight for all positive tumors (*n* = 3) was approximately 81 mg (range, 13–221 mg). Whole body microPET scans were obtained at 72, 96, and 120 hours after injection. MicroCT was obtained at 120 hours only. Figure 3 illustrates a representative image of one animal at each of the different time points. Images shown indicate specific uptake of the radiolabeled anti-CA19-9 antibody on the left shoulder of the mouse where positive xenografts were grown. There is a decreasing amount of background activity visualized by microPET at the later time points. In addition, Figure 3 shows the average percent of injected dose per gram of tissue (%ID/g) for blood, positive tumor, and negative tumor to provide objective confirmation of the radioactivity giving rise to the microPET images. Average blood %ID/g was 0.16 (±0.02) with an average negative tumor %ID/g of 0.04. The %ID/g for each positive tumor was 0.45, 0.54, and 1.43 with an average of 0.80. The average tumor to blood (%ID/g) ratio was 5.0 (*n* = 3), and average positive tumor to negative tumor (%ID/g) ratio was 20.0 (*n* = 1). We found the difference between the average tumor and average blood in the 3 animals to trend toward statistical significance (*P* = 0.056). 

### 3.5. Serum CA19-9 Measurement

Serum CA19-9 measurement in mice harboring the antigen-positive xenograft (BxPC3) or antigen-negative xenograft (MiaPaca-2) with tumors similar in size to those mice used for imaging experiments (7–10 mm). BxPC3 xenograft mice had an average CA19-9 level of 45 units/mL (range of 25–65 units/ml), while MiaPaca-2 xenograft mice demonstrated less than 5 units/mL of CA19-9 in the blood. 

## 4. Discussion

Pancreatic adenocarcinoma is one of the most lethal cancers with a poor overall survival. Most patients have metastatic disease at the time of presentation. For patients who present without evidence of metastatic disease, survival outcomes are only marginally better secondary to the presence of micrometastatic disease, which is not detected by our current imaging modalities (CT/MRI/FDG-PET). Cure is only achieved in the small fraction of patients that undergo surgical resection and have no metastatic or micrometastatic disease. Thus, the vast majority of patients with pancreas cancer undergo chemotherapy with inherent systemic toxicity and limited efficacy. Most recent randomized chemotherapy trials in stage IV pancreas cancer report survival benefit in the range of 1–4 months [27, 28]. These data indicate the need for development of novel agents to specifically target pancreas cancer for both imaging and therapy. 

Since the advent of hybridoma technology approximately 30 years ago, the use of antibodies to treat as well as diagnose a variety of diseases has grown tremendously. Currently, antibody pharmaceuticals represent one of the fastest growing classes of therapeutics in the biotechnology industry [29]. Traditionally, antibodies have been used for *ex vivo* immunohistochemistry of tissues for cancer diagnosis. The murine monoclonal antibody N19-9, which recognizes the tumor antigen CA19-9, has been used over the last 25 years for testing patients' sera and histologic sections of pancreatic adenocarcinomas for the presence of CA19-9 in the diagnosis of pancreas cancer [13, 30]. Based on its utility in evaluating the presence of CA19-9 on *ex vivo* tissues specimens, we sought to investigate the potential to target CA19-9 *in vivo* for PET imaging of pancreas cancer with a radiolabeled anti-CA19-9 antibody. 

CA19-9, also referred to as sialyl Lewis a, is known to be abundantly expressed in >90% of pancreatic cancer cases. The increased expression of CA19-9 is related to aberrant glycosylation of membrane proteins upon malignant transformation of normal pancreatic ductal epithelial cells [20–22]. This aberrant glycosylation has been reported to be secondary to downregulation of certain glycosyltransferases resulting in truncated glycosylation products such CA19-9 [20, 21]. In contrast, CA19-9 is expressed minimally on normal pancreaticobiliary epithelial cells. Moreover, its limited expression is restricted to the apical brush border which is a relatively inaccessible location to targeting agents secondary to tight junctions of mature and polarized ductal epithelia [31]. Upon malignant transformation, epithelial cells lose polarity and glycosylated membrane proteins are no longer exclusively restricted to only a portion of the cell membrane [31]. Therefore, not only does malignant transformation of a pancreas epithelial cell yield aberrant glycosylation and increased production of CA19-9, but also leads to increased accessibility of this antigen.

As a carbohydrate tumor antigen, CA19-9 has a couple of theoretical advantages over conventional protein epitopes in relation to targeting. The first is location of the carbohydrate antigen and the second is the abundance of the epitope. Because CA19-9 is part of a cell's glycan, sugar moieties like CA19-9 are presented at the outermost extent of the cell membrane such that targeting agents such as antibodies can easily access it. Secondly, the epitope can exist in numerous copies per protein resulting in a significant increased presentation of antigen compared to other protein antigens such as CEA. For these reasons, CA19-9 is an attractive antigen target for the development of antibody-based imaging and therapy for pancreas cancer. Our study represents the initial experiments in demonstrating the potential to target CA19-9 with a radiolabeled intact antibody for immunoPET applications. 

First, we sought to characterize and establish a pancreas cancer model with respect to CA19-9 expression. We initially tested our cell lines for expression of CA19-9 qualitatively and determined a high level of expression on the BxPC3 cancer cell line and no expression on the MiaPaca-2 cell line. We then quantified CA19-9 expression on these cell lines and found 1.3 × 10^6^ CA19-9 antigens per BxPC3 cell. For comparison, we tested the well-known high expressing human colon cancer cell line LS174T for carcinoembryonic antigen (CEA) and found approximately 130,000 CEA antigens per cell (data not shown). Based on these results, CA19-9 antigen density per cell is roughly 10x as abundant as CEA indicating a potential advantage as an antigen for targeting. To further characterize our model, we performed IHC on human pancreas cancer specimens as well as our BxPC3 xenograft and found similar staining intensity for CA19-9. Additionally, in order to confirm previous reports that over 90% of pancreas cancers actually express CA19-9, we evaluated 14 surgically resected human pancreas cancer specimens for the presence of CA19-9 by IHC. Of the 14 specimens, 12 showed moderate to strong staining for CA19-9, consistent with results described in the literature [13, 14]. Finally, to further validate our pancreas cancer xenograft model, we tested the blood of mice carrying the antigen-positive xenograft as well as the blood of mice with the antigen-negative xenograft. We determined that the average blood CA19-9 level for mice with the antigen-positive xenograft was 45 units/mL and less than 5 units/mL for mice with antigen-negative xenografts. This demonstrates shedding of CA19-9 into the blood as observed in patients with pancreas cancer. Having characterized our preclinical xenograft model and shown that it is similar to the human cases of pancreas cancer with respect to CA19-9 expression, we hypothesized that we could use radiolabeled anti-CA19-9 monoclonal mouse antibody to achieve antigen-specific targeting of a CA19-9-positive mouse xenograft for PET imaging.

After successfully purifying and radiolabeling the N19-9 antibody with the positron emitting isotope (Iodine-124), it was injected into the tail vein of mice harboring a CA19-9-positive and -negative xenograft such that each mouse served as its own control. Given the anticipated long serum persistence of the intact murine antibody in a mouse, we empirically choose our initial microPET imaging to be performed at 72 hours. Even though the background signal was still significant at 72 hours, we were able to discern that the radiolabeled anti-CA19-9 antibody was preferentially targeting the tumor. Based on these initial microPET images, we decided to image at 96 and again at 120 hrs after injection. Our microPET images at 120 hours were so convincing of antigen-specific tumor targeting with low background activity that we choose to perform the microCT scan and terminate the experiment. The animals were euthanized immediately after the microCT scan and the tumors and blood were harvested and the radioactivity was counted in order to determine the percentage of injected dose per gram of tissue to provide objective evidence that our radiolabeled anti-CA19-9 antibody preferentially targeted the CA19-9-positive tumor. The tumor to blood ratio of 5.0 represents 5 times more radioactivity at the tumor site compared to the blood, which provided an adequate amount of signal contrast between the tumor tissues and background (i.e., blood) to generate our anti-CA19-9 microPET images. Additionally, the positive tumor to negative tumor ratio was 20, signifying specific targeting of the anti-CA19-9 antibody to the antigen-positive tumor instead of nonspecific tumor accumulation through other mechanisms such as the phenomenon known as enhanced permeability and retention [32]. These results provide promising evidence of the utility of CA19-9 as a target for imaging pancreatic cancer.

Although we were able to achieve antigen-specific microPET imaging of mouse xenografts with the anti-CA19-9 antibody, there are some limitations and hurdles that must be overcome before translation into clinical practice. First, the large size of the intact antibody is not ideal for imaging purposes [33]. The long serum half-lives of intact antibodies (10–20 days) cause an extended serum persistence resulting in high background signal and thus reduced contrast for imaging [34]. Although we were able to achieve significant contrast using an intact antibody after 5 days in the mouse, practical imaging applications for humans would most likely need to conform to a time frame of 24 hours or less after injection of an imaging agent. Alternatively, antibodies with shorter half-lives ranging from 2 hours to 10 days can be engineered to fit the purpose of imaging. In particular, Williams et al. found that the ideal same-day imaging agent would be the diabody (scFv dimer; 55 kDa) when compared to the intact antibody, F(ab′)_2_ fragment (110 kDa), and minibody (80 kDa) [35]. Moreover, the intact anti-CA19-9 antibody is a murine monoclonal antibody and would initiate a human anti mouse antibody (HAMA) response if administered to humans, preventing subsequent administrations of the antibody. In order to enable translation into humans, a chimeric antibody at the very least, with human constant regions, would need to be created. In light of these known limitations, we are currently working to create an armamentarium of engineered chimeric anti-CA19-9 antibody fragments with different sizes and pharmacokinetics so that we may select the most suitable fragment for imaging and/or therapy.

In summary, novel molecular imaging techniques such as ImmunoPET imaging have the potential to provide a more clear assessment of the true burden of disease for pancreatic cancer patients. Furthermore, noninvasive imaging as a bridge to targeted therapy also has obvious advantages over our current delivery and evaluation of nontargeted chemotherapeutics. CA19-9 is expressed on almost all pancreas cancers and thus is a potential target for antibody-based molecular imaging or therapy of pancreas cancers. In this study, we characterized a pancreas cancer xenograft model, which recapitulates human pancreas cancer with respect to the antigen CA19-9 expression. Utilizing this model we demonstrated the ability to target CA19-9 with a radiolabeled anti-CA19-9 antibody and produced antigen-specific microPET images. Future studies investigating the utility of engineered chimeric antibody fragments are currently underway. Through these studies, we hope to develop anti-CA19-9-based tumor targeting agents that can be clinically translated for not only novel imaging techniques but also as targeted therapeutics in the fight against pancreas cancer.
